# The impact of APOE genotype, age, and sex on gut microbiota in a mouse model of Alzheimer’s Disease: an exploration of their interactions

**DOI:** 10.1186/s13293-026-00905-w

**Published:** 2026-04-19

**Authors:** Xue Mi, Yi-Chi Zhang, Xu-Jun Zhang, Hao-Bing He, Xiao-Chun Chen, Xiao-Man Dai

**Affiliations:** 1 Public Technology Service Center, Fujian Medical University, Fuzhou, 350122 China; 2 School of Basic Medical Sciences, Fujian Medical University, Fuzhou, 350122 China; 3 Institute of Neuroscience, Fujian Medical University, Fuzhou, 350001 China; 4 Department of Neurology, Fujian Key Laboratory of Molecular Neurology and Institute of Neuroscience, Fujian Medical University Union Hospital, Fujian Medical University, Fuzhou, 350001 China

**Keywords:** Alzheimer’s disease, APOE genotype, Gut microbiota, Age, Sex

## Abstract

**Background:**

As an important interface between the peripheral environment and the central nervous system, the gut microbiota varies greatly between patients or animals with Alzheimer’s disease (AD) and their respective non-AD counterparts; however, it remains unexplored whether the apolipoprotein E (APOE) genotype, age, and sex may interactively influence the characteristics of gut microbiota in AD animals.

**Methods:**

APOE genotype, age, and sex were enrolled as independent variables, with genotype distinguished into APOE3 and APOE4, age into 3 and 10 months, and sex into female and male. The composition, structure, and potential functions of gut microbiota were systematically analyzed by 16S rRNA gene amplicon sequencing to evaluate the individual and interactive effects of APOE genotype, age and sex.

**Results:**

Significant interactions were observed among APOE genotypes, ages, and sexes, with different factor combinations exhibiting distinct effect on microbiotic composition and functional potential. APOE genotype exerted the most significant influence on gut microbiota, followed by age and sex with a relatively minor effect, highlighting the dominant role of host genetic background. Functional prediction analysis indicated that the functional profiles were mainly concentrated in basic metabolic pathways, including the biosynthesis of secondary metabolites and amino acids, and carbon metabolism.

**Conclusion:**

APOE genotype, age, and sex are jointly associated with the structure and potential function of the gut microbiota in AD model mice. These findings provide a perspective of multi-factor interaction into the alterations in gut microbiota in AD and offer new microecological evidence for understanding APOE4-related AD susceptibility, as well as a conceptual basis for future stratified microecological intervention studies.

**Supplementary Information:**

The online version contains supplementary material available at 10.1186/s13293-026-00905-w.

## Abstract (plain language summary)

Alzheimer’s disease (AD) is a common brain disorder that causes various cognitive problems, especially in elderly adults. The APOE gene may affect a person’s risk of developing AD, with one form of this gene (APOE4) indicating a higher risk. Meanwhile, growing evidence suggests that the bacteria living in our gut may influence brain health. However, it remains unclear how genetic background, age, and biological sex work together to shape gut bacteria in AD.

This study used mice of both sexes with different APOE genetypes (APOE3 or APOE4) at different ages to examine the types of gut bacteria present and their potential biological functions. We found that rather than acting alone, these factors showed combined effects on the composition and potential activity of gut bacteria. Different combinations of genetype, age, and sex were associated with distinct gut microbiota patterns. We also found that the predicted functions of gut bacteria varied across these groups, especially in processes related to basic metabolism. These results suggest that genetic risk, age, and sex together shape the gut environment in ways that may be relevant to AD vulnerability.

The findings highlight the importance of considering age and sex when studying genetic risk for AD and suggest that future gut-targeted prevention or intervention strategies may need to be tailored to individual biological backgrounds.

**Supplementary Information:**

The online version contains supplementary material available at 10.1186/s13293-026-00905-w.

## Highlights

APOE genotype, age, and sex may significantly impact the composition of gut microbiota and their predicted functions in an AD mouse model.

APOE genotype, age, and sex may shape gut microbial structure synergistically rather than independently.

APOE genotype exerts the most significant influence on gut microbiota, followed by age and sex with a relatively minor effect, highlighting the dominant role of host genetic background.

Sex is an important biological variable, suggesting its potential influence on microbiota composition and functional characteristics.

**Supplementary Information:**

The online version contains supplementary material available at 10.1186/s13293-026-00905-w.

## Introduction

As the most common neurodegenerative disease, Alzheimer’s disease (AD) is characterized by progressive cognitive decline and memory impairment [1]. With the advancing aging of the global population, AD is increasingly prevalent and has become the main type of dementia in the elderly, posing a serious public health burden [2, 3]. However, the pathogenesis of AD is multi-factorially complex, involving genetic susceptibility, the aging process, sex differences, and environmental factors. Currently, there are no effective disease-modifying treatments [4, 5].

Recently, in the development of AD, the gut-brain axis, especially the role of gut microbiota, has received increasing attention. Studies have documented that gut microbiota can participate in the regulation of central nervous system homeostasis by modulating host immune responses [6], influencing blood-brain barrier permeability [7–9], and producing various neuroactive metabolites (such as short-chain fatty acids, SCFAs) [10–12]. A growing number of clinical and animal studies have shown that gut microbiota dysbiosis is prevalent in patients with AD and AD animal models, manifested as a reduction in beneficial commensal bacteria, an increase in potentially pro-inflammatory bacteria, and abnormal metabolic function [5, 13, 14]. These findings suggest that gut microbiota may serve as an important bridge connecting the peripheral environment and degenerative changes in the central nervous system.

Among the genetic risk factors for AD, the apolipoprotein E (APOE) gene is currently recognized as the gene most closely associated with sporadic AD [15]. Specifically, the APOE4 allele significantly increases the risk of AD and accelerates the age of disease onset [16]. In addition to regulating Amyloid-β (Aβ) deposition, APOE4 is also closely related to enhanced neuroinflammatory responses [17], abnormal lipid metabolism [18, 19], and immune dysfunction [20]. Importantly, recent studies have found that the APOE genotype not only affects the central nervous system but also modulates peripheral immune status and gut microbiota composition, suggesting that APOE4 may participate in the pathological process of AD through the gut-brain axis pathway [21–23].

Apart from the genetic factor, aging is the most important non-genetic risk factor for AD [24, 25]. Epidemiological studies suggest that the risk of developing AD increases significantly with age: after the age of 65, the risk of AD roughly doubles every 5 years; the incidence rate is much higher in the elderly population over 80 years of age than in younger elderly populations [26]. Concurrently, aging is accompanied by profound alterations in the gut microbiota, including reduced microbial diversity, shifts in microbial composition toward potentially pathogenic taxa, impaired intestinal barrier integrity, and functional decline. These age-related microecological changes may promote inflammation and immunosenescence, thereby disrupting the homeostasis of the host immune system [27]. In turn, such inflammatory states may influence the central nervous system through the gut-brain axis, indirectly contributing to the severity of age-related diseases such as cognitive decline [28, 29].

Furthermore, sexual differences also play a significant role in AD. Epidemiological studies show that the prevalence of AD is significantly higher in women than in men, a difference particularly pronounced in APOE4 carriers [30, 31]. The disparity may be accounted for by the changes in estrogen levels, sex-specific immune responses, and metabolic differences [32]. Simultaneously, sex is also an important biological factor influencing the composition and function of the gut microbiota, with significant differences in the composition, abundance, and diversity of the gut microbiota between different sexes. These differences may be regulated by sex hormones (such as estrogen and testosterone) and other physiological factors [33, 34]. Metagenomic analyses of human cohorts have further confirmed that sex-specific gut microbiota characteristics are associated with AD pathogenesis [35]. In transgenic AD mouse models, male mice have been reported to exhibit more pronounced gut microbiota dysbiosis than females [36]. Moreover, microbiota-targeted interventions, including probiotics and antibiotics, have shown significant sex-specific effects on gut microbiota regulation and disease-related phenotypes in male and female AD mice [37]. However, the mechanisms by which sex differences modulate the association between gut microbiota and AD risk, particularly under the APOE4 genetic background, remain insufficiently understood.

Available literature has explored the association of gut microbiota with APOE genotype, sex, or age. In AD transgenic mouse models, the interaction between APOE genotype and sex has been found to significantly influence gut microbiota composition, with a more significant correlation between gut microbiota characteristics and AD pathological manifestations in female APOE4 mice [38]. Age-dependent alterations in gut microbiota have also been reported in APOE mouse models. Comparisons between young and aged APOE3 and APOE4 mice revealed that aging is associated with reduced microbiota diversity and extensive remodeling of the microbiota community structure, effects that are further exacerbated in the APOE4 background, suggesting that APOE genotype may regulate the trajectory of age-related gut microbiota changes [21]. In addition, studies in middle-aged individuals have demonstrated an association between the gut microbiota characteristics in APOE4 carriers and Aβ accumulation in the brain [23], further suggesting that the gut microbiota may partial mediate the influence of APOE4 on AD pathology. Meanwhile, other studies have revealed that age and sex themselves can lead to significant differences in gut microbiota composition and that these differences are closely related to pathological changes in the brain and cognitive decline [35]. Despite these advances, systematic research is scarce to explore the interactive effects of APOE genotype, age, and sex on gut microbiota in a single experimental framework, which, to some extent, limits our comprehensive understanding of the multifactorial synergistic effects of the gut-brain axis in AD pathology.

In summary, although existing studies have highlighted the potential roles of APOE4, age, sex, and gut microbiota in AD, a systematic investigation integrating the interactive effects of APOE genotype, age, and sex on gut microbiota structure and function within the same experimental framework is still lacking. Therefore, in this study, APOE3 and APOE4 mice of both sexes at two representative ages (3 months and 10 months) were examined, and the composition and predicted functions of gut microbiota were systematically analyzed. This study aimed to characterize the gut microbiota alterations under the synergistic influence of genotype, age, and sex, thereby providing novel microecological evidence for a deeper understanding of the mechanisms underlying APOE4-associated AD susceptibility. 

## Materials and methods

### Experimental animals

Human APOE targeted replacement homozygous mice on a C57BL/6J background were obtained from Taconic Biosciences (Rensselaer, NY, USA). In these mice, the endogenous murine Apoe gene was replaced with the human APOE3 or APOE4 allele, and the expression of human APOE is driven by the endogenous mouse Apoe promoter [39]. Male and female APOE3 and APOE4 gene knock-in mice of 3 months old and 10 months old were selected and divided into eight experimental groups: APOE3-3-month-male, APOE3-3-month-female, APOE3-10-month-male, APOE3-10-month-female, APOE4-3-month-male, APOE4-3-month-female, APOE4-10-month-male, and APOE4-10-month-female. Mice were housed in separate cages (3–5 mice per cage) in an environment with a constant temperature (22 ± 1 ℃), constant humidity (50 ± 5%), and a light cycle of 12 h light/12 h dark. They accessed standard feed and water freely.

All experimental protocols involving animals were strictly reviewed by the Institutional Animal Care and Use Committee of Fujian Medical University (IACUC FJMU 2025-0353). The experimental operations were strictly observed the European Community’s “Directive on the Care and Use of Laboratory Animals (2010/63/EU)” to ensure the full implementation of animal welfare and experimental ethics requirements.

### Fecal sample collection

The mice were individually placed in a clean cage lined with sterile filter paper for fecal sample collection. The fresh fecal samples (4–5 fecal pellets per animal) were immediately collected after natural defecation, avoiding urine contamination. Only well-formed, intact fecal pellets were included, while samples that were loose, diarrheal, or contaminated were excluded. The filter paper was replaced for each mouse to prevent cross-contamination. The pellets were collected using sterile forceps and placed in enzyme-free centrifuge tubes, quickly frozen in liquid nitrogen, and then stored at -80 °C, avoiding repeated freeze-thaw cycles. All samples were collected between 8:00 and 10:00 AM to minimize circadian variation in the gut microbiota.

### Gut microbiota detection

#### DNA extraction from fecal samples

Genomic DNA was extracted from the fecal samples collected from mice in each group with the FastPure Stool DNA Isolation Kit (Magnetic bead) (T10-100, MJYH, Shanghai, China). The integrity of the extracted genomic DNA was detected by 1% agarose gel electrophoresis, and the DNA concentration and purity were determined using a NanoDrop 2000 (Thermo Scientific, USA).

#### Polymerase Chain Reaction (PCR) amplification

With the extracted DNA as a template, the V3-V4 hypervariable region of the bacterial 16S rRNA gene was amplified by PCR using upstream primer 338F (5’-ACTCCTACGGGAGGCAGCAG-3’) and downstream primer 806R (5’-GGACTACHVGGGTWTCTAAT-3’) with TransStart Fastpfu DNA Polymerase (AP221-02, TransGen Biotech, Beijing, China) [40]. PCR was performed under the following conditions: initial denaturation at 95 ℃ for 3 min; 27 cycles (denaturation at 95 ℃ for 30 s, annealing at 55 ℃ for 30 s and extension at 72 ℃ for 45 s), followed by a final extension at 72 ℃ for 10 min and holding at 4 ℃. PCR products were separated by 2% agarose gel electrophoresis and purified with a DNA gel recovery and purification kit (C01-10000, MJYH, Shanghai, China). The purified PCR products were then quantified with a Qubit 4.0 (Thermo Fisher Scientific, USA).

#### Sequencing library construction

The purified PCR products were used to construct sequencing libraries with the NEXTFLEX Rapid DNA-Seq Kit (NOVA-5144; PerkinElmer, Inc., Austin, USA) according to the manufacturer’s instructions. Briefly, adapter sequences were added to the outer end of the target region by PCR; (2) adapter dimers were removed using magnetic bead-based size selection, and the libraries were enriched by PCR amplification. The final libraries were purified by magnetic beads and subjected to sequencing on the Illumina Nextseq2000 platform (Illumina, San Diego, USA) by Majorbio Bio-Pharm Technology Co. Ltd. (Shanghai, China). The raw sequencing data have been deposited in the National Center for Biotechnology Information (NCBI) Sequence Read Archive (SRA) database and will be made publicly available upon publication.

#### High-throughput sequencing data analysis

All data analysis was performed on the Majorbio Cloud platform (https://cloud.majorbio.com). Specifically, the raw paired-end reads were quality-filtered with fastp (version 0.19.6) [41] and merged with FLASH (version 1.2.7) [42]. The resulting sequences were denoised using the DADA2 plugin [43] within the QIIME 2 workflow (version 2024.10, https://qiime2.org) [44] with default parameters (maxEE = 5, truncQ = 0, and maxN = 0), to generate an amplicon sequence variant (ASV) table. Non-bacterial sequences such as mitochondria and chloroplast were removed prior to downstream analysis. Taxonomic classification of ASVs was performed using the Naive Bayes classifier implemented in QIIME 2 against the SILVA 16S rRNA gene database (v138.2). To minimize the effect of sequencing depth on subsequent diversity analysis, all samples were rarefied to 30,696 sequences based on the minimum sequencing depth across samples [45].

The within-sample α-diversity was evaluated by assessing richness indices (Sobs and ACE), the Shannon diversity index, Pielou’s evenness index, and Good’s sequencing coverage index [46]. All α-diversity metrics were calculated with Mothur (http://www.mothur.org/wiki/Calculators) [47]. The sequencing depth adequacy was examined by rarefaction curves based on the Sobs index. The between-sample diversity (β-diversity) was assessed with multiple distance metrics, including Jaccard distance (presence/absence-based), Bray–Curtis distance (abundance-based), and both weighted and unweighted UniFrac distances (phylogeny-based) [46, 48]. The differences in microbial community structure among groups were visualized by adopting principal coordinate analysis (PCoA) [49] and non-metric multidimensional scaling (NMDS) [50] as complementary ordination methods. The effects of APOE genotype, age, and sex on gut microbiota composition were evaluated by permutational multivariate analysis of variance (PERMANOVA) with 999 permutations. To assess whether observed differences in β-diversity were influenced by within-group dispersion, all distance matrices were analyzed by permutational analysis of multivariate dispersions (PERMDISP) [51]. For enterotype analysis, Jensen–Shannon divergence (JSD) was calculated at the genus level and used as the distance metric for community clustering to identify distinct microbial community types [52]. In addition, hierarchical clustering based on the Jaccard distance matrix was performed to visualize the similarity relationships among samples and to depict the overall structure of microbial community composition. The differences in genus-level abundance were identified using linear discriminant analysis effect size (LEfSe) with LDA > 3 and *p* < 0.05 [53]. Functional prediction of 16S rRNA genes was performed using PICRUSt2 (version 2.2.0) [54].

### Statistical analysis

Data were presented as mean ± SEM and statistically analyzed with GraphPad Prism 9.0 software (GraphPad Prism Software Inc., USA). Data normality was assessed by the Shapiro–Wilk test, and homogeneity of variance was evaluated by Levene’s test to guide the selection of appropriate statistical methods. The Kruskal–Wallis rank-sum test was used for comparisons among multiple groups, and Mann–Whitney U test was used for comparisons between two groups, followed by Dunn’s post hoc test for multiple-group comparisons. P values were adjusted using the false discovery rate (FDR) where applicable. The statistical difference was designated at *p* < 0.05.

## Results

### Differences in the α-diversity of gut microbiota among different groups of mice

**Fig. 1:**
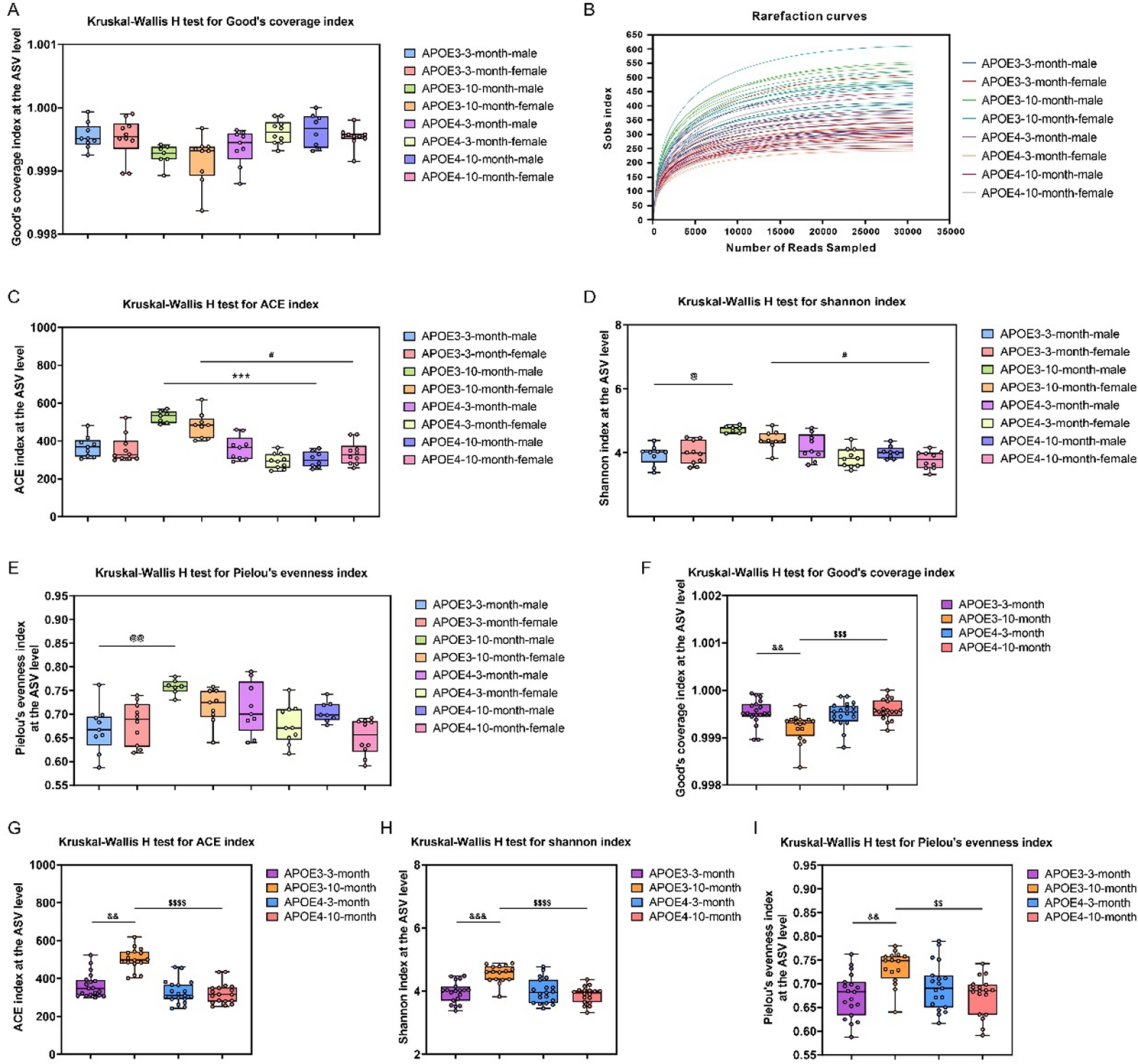
Analysis of α-diversity in the gut microbiota across different groups at the ASV level. (**A**-**E**) α-diversity indices for individual groups: (**A**) Good’s coverage, (**B**) rarefaction curve, (**C**) ACE, (**D**) Shannon, and (**E**) Pielou’s evenness. Data are expressed as mean ± SEM, *n* = 7–10 per group. ^@^ *p* < 0.05, ^@@^ *p* < 0.01, as compared with the APOE3-3-month-male group; *** *p* < 0.001, as compared with the APOE3-10-month-male group; ^#^ *p* < 0.05, as compared with the APOE3-10-month-female group. (**F**-**I**) After merging male and female mice of the same genotype and age: (**F**) Good’s coverage, (**G**) ACE, (**H**) Shannon, and (**I**) Pielou’s evenness. Data are expressed as mean ± SEM, *n* = 16–19 per group. ^&&^ *p* < 0.01, ^&&&^ *p* < 0.001, as compared with the APOE3-3-month group; ^$$^ *p* < 0.01, ^$$$^ *p* < 0.001, *p* < 0.0001, as compared with the APOE3-10-month group

We first compared the coverage of gut microbiota among different groups. As shown in Fig. 1A, the Good’s coverage index approached 1 (ranging from 99.84% to 99.93%) for all groups, indicating near-complete coverage of the gut microbiota community. The rarefaction curves based on the Sobs index at the ASV level (Fig. 1B) showed that the curves for all samples plateaued with increasing sequencing depth, indicating that the sequencing depth was sufficient for subsequent analyses.

We then analyzed the α-diversity in all groups. Microbial richness was assess using the ACE index (Fig. 1C), microbial diversity using the Shannon index (Fig. 1D), and microbial evenness using the Pielou’s evenness index (Fig. 1E). As shown in Fig. 1C, the ACE index was higher in both male and female 10-month-old APOE3 mice than in age-matched APOE4 mice, indicating higher microbial richness (male: *p* = 0.0004; female: *p* = 0.0419). For the Shannon index (Fig. 1D), 10-month-old APOE3 male mice showed the highest microbial diversity. This difference was statistically significant compared with the 3-month-old APOE3 male mice (*p* = 0.0210). Furthermore, the 10-month-old APOE3 female mice exhibited higher diversity than the age-matched APOE4 females (*p* = 0.0241). In the analysis of microbial evenness, 10-month-old male APOE3 mice showed higher evenness, whereas 10-month-old female APOE4 mice exhibited lower evenness. Statistical comparisons revealed that 10-month-old male APOE3 mice had significantly higher evenness than 3-month-old male APOE3 mice (*p* = 0.0070), with greater intra-group variation observed in the latter group (Fig. 1E).

As the above analysis indicated that sex played a relatively minor role, male and female mice with the same genotype and age were pooled for further inter-group comparisons (Fig. 1F-I). In the APOE3 genotype, compared with those of 3-month-old mice, the 10-month-old mice exhibited significantly higher gut microbiota coverage, richness, diversity, and evenness (Good’s coverage: *p* = 0.0040; ACE: *p* = 0.0015; Shannon: *p* = 0.0008; Pielou’s evenness: *p* = 0.0014; Fig. 1F-I). In addition, compared with the age-matched APOE4 mice, the 10-month-old APOE3 mice showed significantly higher gut microbiota coverage (*p* = 0.0008), richness (*p* < 0.0001), diversity (*p* < 0.0001), and evenness (*p* = 0.0015) (Fig. 1F-I). Collectively, these α-diversity analyses indicate that both APOE genotype and age are significantly associated with gut microbiota diversity. With increasing age, the APOE3 mice may maintain higher gut microbiota diversity and richness compared with the APOE4 genotype.

### Differences in β-diversity of gut microbiota among different mouse groups

**Fig. 2:**
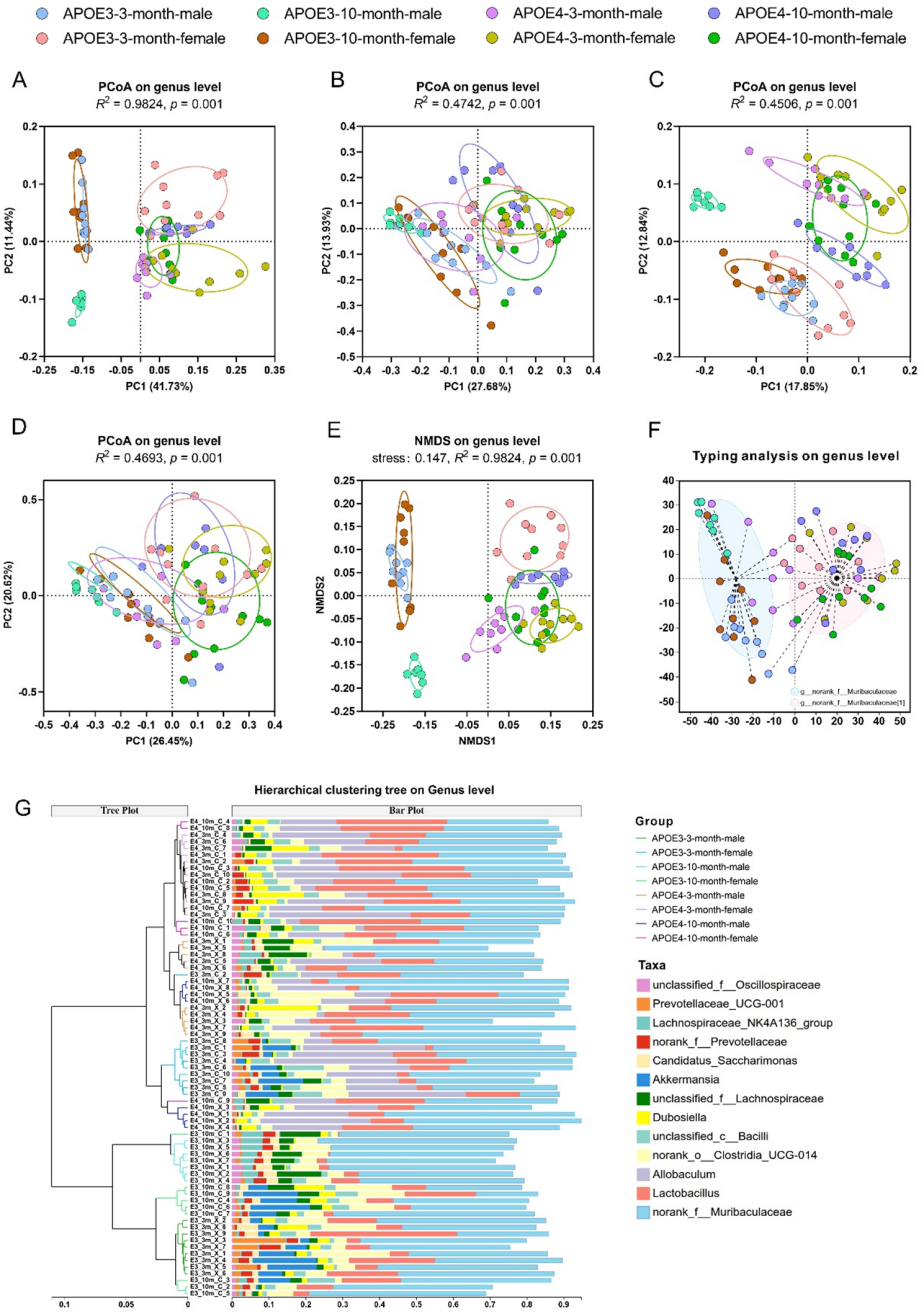
Analysis of gut microbiota β-diversity at the genus level in mice. (**A**-**D**) PCoA based on (**A**) Jaccard distance, (**B**) Bray–Curtis distance, (**C**) unweighted UniFrac distance, and (**D**) weighted UniFrac distance among the eight groups. (**E**) NMDS analysis for gut microbiota β-diversity. (**F**) Enterotype clustering of 72 samples into enterotype 1 (light blue) and enterotype 2 (light pink). (**G**) Hierarchical clustering based on the Jaccard distance. *n* = 7–10 per group

At the genus level, β-diversity was assessed using four distance metrics—Jaccard distance, Bray–Curtis distance, unweighted UniFrac distance, and weighted UniFrac distance—and analyzed by PCoA and PERMANOVA to evaluate the effects of APOE genotype, age, and sex grouping on gut microbiota composition. PERMANOVA results showed that the overall “APOE genotype × sex × age” model exhibited significant differences in gut microbiota β-diversity across all distance metrics (*p* = 0.001). However, the proportion of variance explained (*R*²) differed significantly among the different distance metrics (Fig. 2). When the differences in taxonomic presence/absence were evaluated using the Jaccard distance, the PERMANOVA model explained a large proportion of the variation in community structure (*R*² = 0.9824; Fig. 2A), suggesting that differences in taxonomic composition among samples with different APOE genotypes, ages, and sexes contributed substantially to β-diversity. In contrast, for distance metrics incorporating abundance information (Bray–Curtis distance, *R*² = 0.4742; Fig. 2B), phylogenetic relationships (unweighted UniFrac distance, *R*² = 0.4506; Fig. 2C), or both abundance and phylogeny (weighted UniFrac distance, *R*² = 0.4693; Fig. 2D), the explained variance was markedly lower. Consistently, greater overlap among groups was observed in the corresponding PCoA plots, with residual variation accounting for more than 50% of the total variation (Table 1), suggesting that differences in relative abundance and phylogenetic structure were less pronounced across groups. These results indicate that the observed group differences are primarily driven by the presence or absence of specific taxa rather than large shifts in their relative abundances or phylogenetic composition. The incomplete separation observed in PCoA plots therefore likely reflects the complex and multifactorial nature of gut microbiota variation, with a substantial proportion of variation not fully explained by the grouping factors examined in this study.

**Table 1:** PERMANOVA analysis of gut microbiota β-diversity at the genus level using different distance metrics

Distance metrics	Effect type	df	*R* ^2^	F	Pr(> F)	Significance
Jaccard distance	Model	7	0.98241	510.6600	0.0001	***
	APOE	1	0.39003	1419.1640	0.0001	***
	Sex	1	0.16715	608.1800	0.0001	***
	Age	1	0.11083	403.2780	0.0001	***
	APOE: Sex	1	0.04694	170.7840	0.0001	***
	APOE: Age	1	0.13153	478.5800	0.0001	***
	Sex: Age	1	0.14381	523.2560	0.0001	***
	APOE: Sex: Age	1	–0.00786	–28.6160	1.0000	
	Residual	64	0.01759			
	Total	71	1			
Bray–Curtis distance	Model	7	0.47417	8.2446	0.0001	***
	APOE	1	0.14567	17.7293	0.0001	***
	Sex	1	0.09524	11.5919	0.0001	***
	Age	1	0.03564	4.3384	0.0019	**
	APOE: Sex	1	0.02006	2.4415	0.0342	*
	APOE: Age	1	0.07479	9.1035	0.0001	***
	Sex: Age	1	0.07338	8.9318	0.0001	***
	APOE: Sex: Age	1	0.02938	3.5761	0.0049	**
	Residual	64	0.52583			
	Total	71	1			
Unweighted-Unifrac distance	Model	7	0.4506	7.4988	0.0001	***
	APOE	1	0.17066	19.8801	0.0001	***
	Sex	1	0.03399	3.9590	0.0003	***
	Age	1	0.04834	5.6313	0.0002	***
	APOE: Sex	1	0.03441	4.0088	0.0002	***
	APOE: Age	1	0.08378	9.7601	0.0001	***
	Sex: Age	1	0.03191	3.7171	0.0007	***
	APOE: Sex: Age	1	0.04751	5.5349	0.0001	***
	Residual	64	0.5494			
	Total	71	1			
Weighted-Unifrac distance	Model	7	0.46931	8.0852	0.0001	***
	APOE	1	0.14077	16.9758	0.0001	***
	Sex	1	0.09637	11.6221	0.0001	***
	Age	1	0.03538	4.2663	0.0031	**
	APOE: Sex	1	0.01715	2.0687	0.0757	ns
	APOE: Age	1	0.06368	7.6795	0.0001	***
	Sex: Age	1	0.09358	11.2853	0.0001	***
	APOE: Sex: Age	1	0.02238	2.6989	0.0271	*
	Residual	64	0.53069			
	Total	71	1			

Note: * *p* < 0.05, ** *p* < 0.01, *** *p* < 0.001; non-significant results are indicated by “ns”

To evaluate whether these differences were influenced by dispersion effects, PERMDISP analysis was performed. No significant differences in dispersion were observed among groups for Jaccard (*p* = 0.094), Bray–Curtis (*p* = 0.118), or weighted UniFrac distances (*p* = 0.076). A marginally significant dispersion difference was detected only for the unweighted UniFrac distance (*p* = 0.044) (Table S1). These results suggest that the observed β-diversity differences are primarily attributable to compositional variation rather than dispersion effects.

Further analysis of the main effects and interaction effects revealed that APOE genotype was the single factor most strongly associated with gut microbiota structure across the different distance metrics, although its explanatory power varied markedly among dimensions (Table 1). Under the Jaccard distance metric, the proportion of variance in gut microbiota composition explained by APOE genotype (*R²* = 0.3900) was substantially higher than that explained by sex (*R*² = 0.1672) or age (*R*² = 0.1108). In contrast, under the Bray–Curtis (*R*² = 0.1457), weighted UniFrac (*R²* = 0.1408), and unweighted UniFrac distance metrics (*R*² = 0.1707), the variance explained by APOE genotype was significantly reduced, and the main effects of sex and age were further weakened (*R*² < 0.1). The analysis of the interaction effect showed that the APOE genotype × age and that of sex × age interactions contributed to gut microbiota variation across multiple distance metrics. Under the Jaccard distance metric, the proportion of variance in gut microbiota composition explained by these two interaction terms were *R*² = 0.1315 and *R*² = 0.1438, respectively, and both showed significant contributions under Bray–Curtis, unweighted UniFrac, and weighted UniFrac distance metrics (*p* < 0.01). Notably, the explanatory power of sex × age interaction under the weighted UniFrac distance (*R*² = 0.0936) was comparable to that of the main effect of sex. In contrast, the three-way interaction (APOE genotype × sex × age) showed only weak significance in some distance metrics (e.g., weighted UniFrac, *p* = 0.0271), with explained variance below 0.05, indicating that its biological relevance was limited.

Subsequently, non-metric multidimensional scaling (NMDS) based on the Jaccard distance metric was performed to further validate the observed grouping patterns in gut microbiota structure. As shown in Fig. 2E, samples grouped by the combination of APOE genotype, age, and sex exhibited clear aggregation and separation, with a stress value of 0.147 (< 0.2), indicating that the ordination results were reliable. PERMANOVA analysis further demonstrated that this grouping explained a substantial proportion of the variation in gut microbiota structure (*R*² = 0.9824, *p* = 0.001), which was consistent with the PCoA results based on the Jaccard distance metric. Collectively, these results indicate that similar grouping patterns can be observed using different ordination approaches, and highlight the importance of taxonomic presence/absence patterns in shaping gut microbiota differences among groups.

Given the JSD-based unsupervised clustering of gut microbiota at the genus level (Fig. 2F), all samples were classified into two distinct community subtypes dominated by *g_norank_f_Muribaculaceae*, designated as Type 1 and Type 2. Although both subtypes were dominated by *g_norank_f_Muribaculaceae*, they were distinguished by distinct abundance distribution patterns of this dominant taxon and associated genera. Samples belonging to Type 1 were predominantly derived from APOE3 mice, whereas Type 2 samples were mainly associated with APOE4 mice, suggesting a genotype-associated differentiation within *Muribaculaceae*-dominated microbiota structures. Notably, partial cross-group mixing was observed among 3-month-old samples, indicating that age may modulate the APOE genotype-associated differentiation patterns. Furthermore, hierarchical clustering of genus-level microbial profiles revealed an initial separation by APOE genotype, followed by further stratification by age and sex within each genotype (Fig. 2G). A small number of samples showed cross-boundary clustering, which was consistent with the PCoA results and the observed interaction effects.

Altogether, the above results suggest a dimension-specific pattern in the associations of APOE genotypes, sex, and age with the gut microbiota variation. Differences based on taxonomic presence/absence, as captured by Jaccard-based analyses, appeared to contribute most strongly to group differentiation, with APOE genotype showing the strongest association among the examined factors. The effects of APOE genotype and sex on gut microbiota structure exhibited age-dependent patterns. In contrast, analyses incorporating species abundance or phylogenetic relationships showed weaker group separation, indicating a relatively smaller contribution of these dimensions to the observed microbiota differences.

### Taxonomic composition and differences in microbial communities at the genus level

**Fig. 3:**
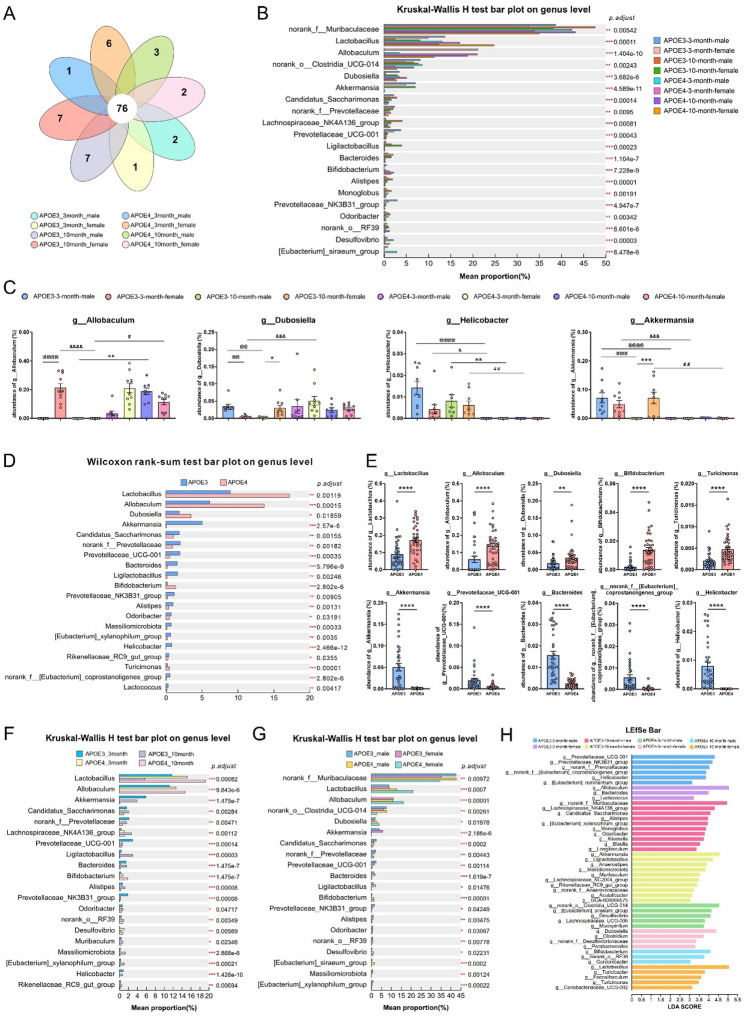
Species composition and differential analysis at the genus level across the eight groups. (**A**) Venn diagram of species composition. (**B**) Top 20 abundant species with differences across groups (*n* = 7–10 per group). (**C**) Relative abundance of *Allobaculum*, *Dubosiella*, *Helicobacter*, and *Akkermansia*. Kruskal–Wallis rank-sum test was used for comparisons. Data are expressed as mean ± SEM, *n* = 7–10 per group. ^@@^ *p* < 0.01, ^@@@^ *p* < 0.001, ^@@@@^ *p* < 0.0001, as compared with the APOE3-3-month-male group; ^&^ *p* < 0.05, ^&&&^ *p* < 0.001, ^&&&&^ *p* < 0.0001, as compared with the APOE3-3-month-female group; ^*^ *p* < 0.05, ^**^ *p* < 0.01, ^***^ *p* < 0.001, as compared with the APOE3-10-month male group; ^#^ *p* < 0.05, ^##^ *p* < 0.01, as compared with the APOE3-10-month-female group. (**D**) Difference analysis after merging mice with the same genotype. Top 20 differentially abundant species are shown. (**E**) Abundance of *Lactobacillus*, *Allobaculum*, *Dubosiella*,* Bifidobacterium*, *Turicimonas*, *Akkermansia*, *Prevotellaceae_UCG-001*, *Bacteroides*, *g_norank_f__[Eubacterium]_coprostanoligenes_group*, and *Helicobacter* in the two groups. Wilcoxon rank-sum test was used for comparison between the two groups. Data are expressed as mean ± SEM, *n* = 35 for APOE3 group, *n* = 37 for APOE4 group; ^**^ *p* < 0.01, ^****^ *p* < 0.001, as compared with the APOE3 group. (**F**-**G**) Male and female mice of the same genotype and age (**F**) or the same genotype and sex (**G**) were combined before the microbial community difference analysis. Top 20 differentially abundant species are shown. Kruskal–Wallis rank-sum test was used for the abundance comparison in the groups. (**H**) LefSe analysis of the eight groups at the genus level, LDA score (log 10) > 3.0, *p* < 0.05

We analyzed the taxonomic composition of the microbial communities in the eight groups at the genus level and evaluated the inter-group differences. The Venn diagram of microbial taxa (Fig. 3A) revealed 76 core shared taxa among the eight groups, accounting for the majority (72.38%) of the total detected taxa. Each group harbored 1–7 group-specific taxa, with the APOE3-10-month-female and APOE3-10-month-male groups each containing 7 unique taxa, and the APOE4-3-month-female group containing 6 unique taxa. These results are consistent with the Jaccard distance-based results mentioned above, suggesting that differences in group-specific taxa contribute to the differentiation of microbial community structure. We then analyzed the differential microbial communities among the eight groups. The Kruskal–Wallis H test combined with FDR correction identified significant abundance differences among the 85 gut microbiota taxa at the genus level across the eight groups (*p.adjust* < 0.05; Table S2). Among the top 20 most abundant taxa shown in Fig. 3B, *g_norank_f_Muribaculaceae*, *Lactobacillus*, and *Allobaculum* exhibited the most pronounced differences. In addition, the relative abundance of *Allobaculum*, *Dubosiella*, *Helicobacter*, and *Akkermansia* differed significantly among groups (Fig. 3C).

We then used the Mann–Whitney U test to identify 55 significantly differentially abundant genera between APOE3 and APOE4 mice (Table S3; Fig. 3D). Among the differentially abundant genera, *Lactobacillus*, *Allobaculum*, and *Dubosiella* were the most abundant and showed higher relative abundance in APOE4 mice compared to APOE3 mice. *Akkermansia* and *Helicobacter* were predominantly found in APOE3 mice (Fig. 3E). Other genera such as *Bifidobacterium* and *Turicimonas* were more abundant in APOE4 mice, whereas *Prevotellaceae_UCG-001*, *Bacteroides*, and *g_norank_f__[Eubacterium]_coprostanoligenes_group* were less abundant in APOE4 mice compared to APOE3 mice (Fig. 3E).

In the interaction analysis, as shown in Table S4, a total of 73 significantly differentially abundant genera were identified among the four groups of APOE × age. Among them, *Prevotellaceae_UCG-001* and *Prevotellaceae_NK3B31_group* were most abundant in the APOE3 3-month-old mice, whereas *Desulfovibrio* was present at lower abundance in this group compared to others. *Ligilactobacillus*, *Lachnospiraceae_NK4A136_group*, *Massiliomicrobiota*, and *[Eubacterium]_xylanophilum_group* were mainly enriched in the APOE3 10-month-old mice, while *Bifidobacterium* was largely absent in this group (Fig. 3F). In APOE4 10-month-old mice, *Lactobacillus* and *Allobaculum* were the most abundant differentially abundant genera. Among the 64 significantly differentially abundant genera across the four APOE genotype × sex groups (Table S5), *g_norank_f_Muribaculaceae* and *Lactobacillus* were the most abundant. Certain taxa were present at low levels in specific groups; for example, *Allobaculum* was almost unexpressed in the APOE3 male group; *g_norank_f__Prevotellaceae* in the APOE4 males; *Bifidobacterium* and *Desulfovibrio* in the APOE3 females; *Akkermansia* and *Bacteroides* in APOE4 mice of both sexes; and *g_norank_f_Muribaculaceae* and *g__norank_o__Clostridia_UCG-014* exhibited sex-specific differences only in APOE4 mice, but not in APOE3 mice (Fig. 3G). These differences in microbial taxa abundance were consistent with the PERMANOVA results, indicating that APOE genotype showed the strongest association with microbiota structure, APOE genotype × age represented the most pronounced interaction, and sex acts as a secondary modifier. Together, these three factors were associated with the observed group-specific abundance patterns of the gut microbiota.

To identify the core taxa biomarkers specific to each group based on APOE genotype × age × sex, LEFSe analysis (LDA score ≥ 3.0; *p* < 0.05) were performed on the differentially abundant genera identified by the Kruskal–Wallis test. The results showed that a total of 46 genus-level taxa biomarker were identified across the eight groups, with no duplicates (Fig. 3H), indicating high specificity of APOE genotype, age, and sex in shaping gut microbiota composition. The APOE3 10-month-old male and female mice each had 10 biomarkers, whereas the APOE3 3-month-old females and APOE4 10-month-old males had fewer biomarkers (3 each).

### Functional predictive analysis

**Fig. 4:**
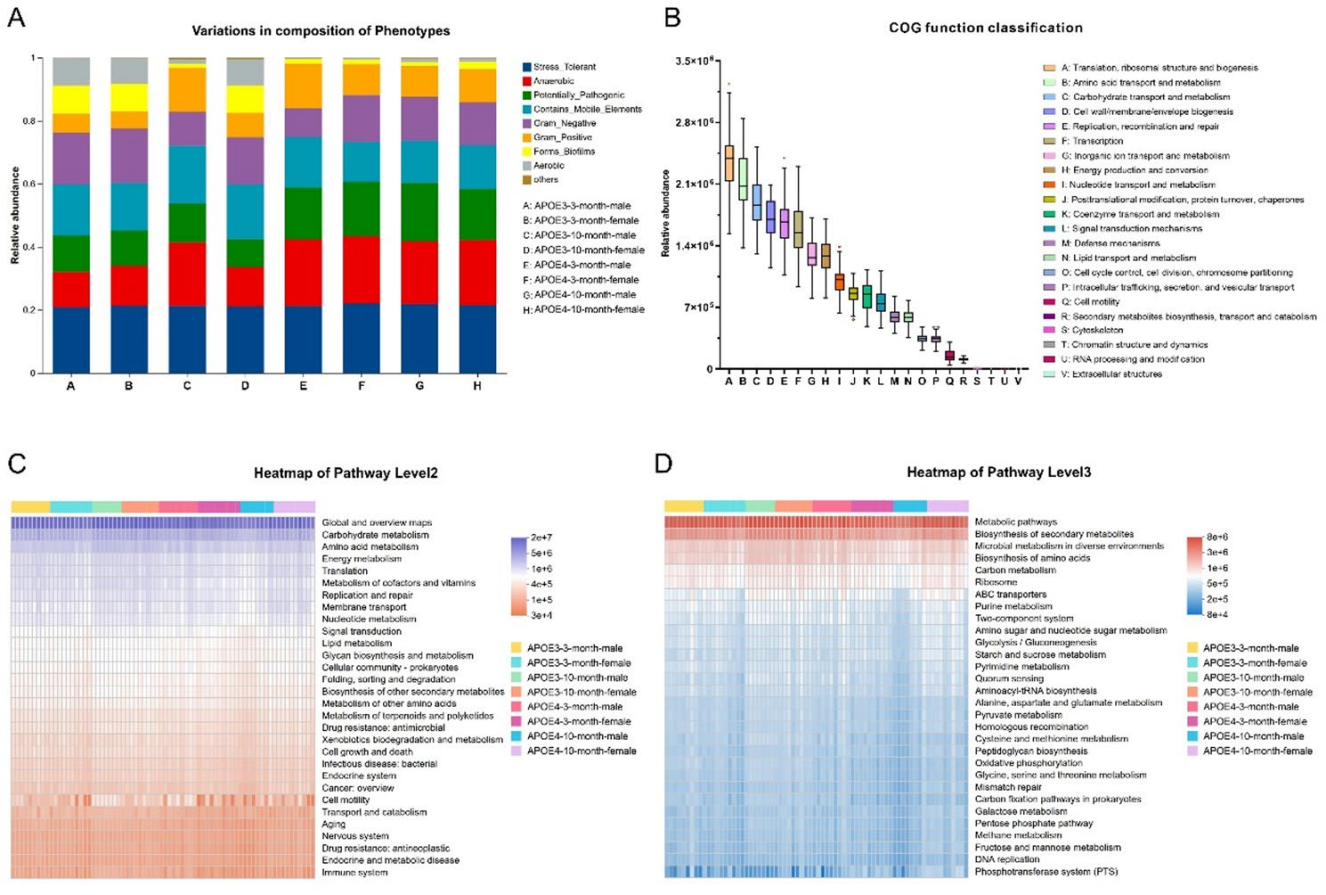
Functional prediction analysis of bacterial microbiota. (**A**) BugBase-based prediction of eight gut microbiota phenotypes: Gram-positive, Gram-negative, biofilm-forming, potentially pathogenic, mobile element-containing, aerobic, anaerobic, and oxidative stress-tolerant. (**B**) Functional prediction using PICRUSt2 combined with the egNOG database. (**C**-**D**) Functional prediction using PICRUSt2 combined with the KEGG database, showing KEGG pathway results at level 2 (**C**) and 3 (**D**)

We first employed BugBase to predict the functional phenotypes of the gut microbiota across the eight experimental groups, identifying eight microbial phenotypes, including Gram positive, Gram negative, potentially pathogenic, aerobic, anaerobic, oxidative stress-tolerant, biofilm-forming and mobile elements-containing bacteria (Fig. 4A, Table S6). Among these phenotypes, the relative abundance of the oxidative stress-tolerant bacteria remained stable at approximately 0.2 across all groups, representing a core microbial feature that was not markedly influenced by genotype, age, or sex. Compared with APOE3 mice at the same age, APOE4 mice exhibited a consistently higher proportion of potentially pathogenic phenotypes, accompanied by a lower abundance of biofilm-forming bacteria. In addition, the gut microbiota of the APOE4 mice showed a reduced proportion of aerobic phenotypes and a corresponding increase in anaerobic phenotypes relative to APOE3 mice. Notably, the microbial phenotypes distribution of 10-month-old male APOE3 mice closely resembled that observed in the APOE4 groups. Moreover, within the same genotype and age, male and female mice displayed highly similar functional phenotype profiles.

Subsequently, PICRUSt2-based functional prediction was performed on the 16S rRNA gene amplicon sequencing data and annotated against the eggNOG and KEGG databases to characterize the potential functional profiles of the gut microbiota (Fig. 4B-D). As shown in the eggNOG-based analysis (Fig. 4B, Table S7), the predicted microbial functions were predominantly enriched in translation, ribosomal structure and biogenesis, as well as amino acid and carbohydrate transport and metabolism. These functions primarily represent the fundamental processes that maintain basic cellular life activities.

Consistently, KEGG Level 2 functional annotation indicated that gut microbiota function was mainly associated with the metabolism of carbohydrate, amino acid, and energy (Fig. 4C, Table S8). These core metabolic pathways represented the dominant functional components across all groups, while subtle shifts in their relative contributions may occur under different genotypic, age, and sex conditions, reflecting the overall functional structure under varying biological contexts. In contrast, certain categories related to environmental adaptation and microbial behavior, such as membrane transport, signal transduction, and cell motility, exhibited relatively greater variability in their distribution. Notably, the variability observed in cell motility may be attributed to the fact that this function is primarily associated with a subset of motile bacteria possessing flagella and chemotaxis systems, rather than being universally present across all gut microbes. Therefore, its relative abundance is more sensitive to compositional fluctuations in specific taxa, which may contribute to the observed variation across samples.

Further analysis at the KEGG Level 3 revealed that the enriched pathways were mainly involved in global and overview maps, including metabolic pathways, biosynthesis of secondary metabolites, biosynthesis of amino acids, and carbon metabolism (Fig. 4D, Table S9), highlighting the central role of metabolic processes in shaping the functional potential of the gut microbiota. Taken together, these results suggest that while core metabolic functions remain relatively conserved, their relative contributions, as well as functions related to environmental adaptation and microbial behavior, may exhibit flexible distribution patterns under different conditions.

## Discussion

The relationship between gut microbiota and AD has received increasing attention in the field of neurodegenerative disorders. Accumulating evidence indicates that the gut microbiota composition in patients with AD and individuals with mild cognitive impairment (MCI) differs significantly from that of healthy controls, suggesting that the microbiota may serve as an early biomarker of cognitive decline [55].

In the present study, through a multidimensional and systematic analysis, we demonstrate that APOE genotype, age, and sex exert interactive regulatory effects on both the structure and functional potential of the gut microbiota in mice. Notably, our results further indicate that microbial taxonomic “presence/absence” patterns constitute a key ecological dimension underlying microbiota differentiation. Moreover, we clarify the dominant regulatory role of APOE genotype and identify the APOE genotype × age interaction as a critical modulatory axis shaping gut microbiota composition. Together, these findings provide novel experimental evidence supporting a mechanistic link between the gut microbiota alterations and APOE4-related AD pathogenesis.

### APOE genotype as the primary determinant of gut microbiota structure

The results of this study indicate that APOE genotype has a significant impact on the composition and structure of the gut microbiota. In the α-diversity analysis, the APOE3 genotype maintained a higher microbiota diversity and richness; furthermore, the multidimensional β-diversity analysis showed that the APOE genotype had a relatively higher explanatory power for microbiota variation than sex and age; through the intergroup comparison of microbiota differences, we identified 55 significantly different genus-level taxa between APOE3 and APOE4 mice; and BugBase phenotypic prediction results showed that APOE4 genotype mice exhibited highly consistent microbial functional phenotypes under different ages and sexes, suggesting that APOE4 may function as a dominant genetic factor, substantially shaping the functional state of the gut microbiota. In contrast, the microbiota phenotype of the APOE3 genotype mice was more sensitive to age and sex. These results are highly consistent with previous findings that APOE genotype influences gut microbiome structure in humans and mice [21, 56].

In human studies, APOE ε4 carriers report significant differences in gut microbiota composition and functional characteristics when compared with healthy adults [22]. In an APOE-targeted replacement mouse model, different APOE alleles significantly affect the β-diversity and relative abundance of specific bacterial genera in the gut microbiota. The microbial composition of APOE4 mice differs significantly from that of APOE3 mice, suggesting that the APOE genotype itself may contribute to gut microbiota remodeling [21, 22]. In addition, another study of 6-month-old APOE2, APOE3 and APOE4 genotype homozygous mice (male and female mice) indicates that, regardless of sex, there is significant taxonomic divergence in their gut microbiome [57].

In this study, phenotypic prediction suggested a significantly higher proportion of potential pathogenic bacteria in the APOE4 mice when compared with the APOE3 mice, with *Lactobacillus*, *Allobaculum*, and *Dubosiella* among the genera showing significant enrichment in APOE4 genotype mice. Notably, although *Lactobacillus* is generally considered a commensal or probiotic, numerous clinical case studies have demonstrated its conditional pathogenicity, capable of inducing severe infections in clinical settings, such as bacteremia and endocarditis under conditions of immunodeficiency or impaired intestinal barrier [58, 59]. *Allobaculum* species have been reported to possess mucin-degrading capabilities, invading or altering the intestinal mucosa and potentially disrupting local barriers under conditions of imbalance [60], and display potential pro-inflammatory properties in colitis and immune stimulation models [61]. Its enrichment in APOE4 mice may be associated with altered intestinal barrier function or immune responses. *Dubosiella* has been thought in recent years to promote the abnormal accumulation of branched-chain amino acids and enhance the spread of α-synuclein pathology, thus promoting the neurodegenerative process of Parkinson’s disease [62]. In the colitis model, its abundance changes coexist with inflammatory imbalance, suggesting that it may be involved in inflammatory microecological disturbances [63]. Its elevation in APOE4 mice may reflect the metabolic and inflammatory susceptibility associated with the APOE4 genotype.

In contrast, *Akkermansia*, which is significantly enriched in APOE3 mice, is widely considered to be closely related to intestinal barrier integrity and metabolic homeostasis [64–67]. Its significant reduction in APOE4 mice may suggest a potential association with impaired mucus layer homeostasis or anti-inflammatory regulation in APOE4 mice. Furthermore, the significant reduction of *Prevotellaceae* (including *Prevotellaceae_UCG-001*) and *Bacteroides* in the APOE4 mice suggests that the APOE4 genotype may be associated with weakened capacity for complex carbohydrate degradation and the production of SCFAs [68, 69]. This alteration may affect host metabolism and the inflammatory microenvironment, further impacting immune homeostasis and the intestinal barrier.

Altogether, our findings support a close link between APOE genotype and gut microbiota composition, and reveal that APOE4 mice exhibit significantly different microecological characteristics when compared with APOE3 counterparts. These differentially expressed microbiota may collectively participate in APOE genotype-related physiological or pathological processes by regulating intestinal barrier function, immune responses, and metabolic pathways. However, it is noteworthy that the central regulatory role of APOE genotype does not exist independently, but rather through dynamic regulation in interaction with age and sex, for the intensity and specific characteristics of microbiota differences differ significantly among individuals with the same genotype at different ages and sexes. This provides a microecological perspective for understanding individual differences in APOE-related AD cases.

### Age-dependent and APOE genotype-specific modulation of the gut microbiota

Age, as a key natural factor in gut microbiota remodeling, exhibits significant stage-specific regulation of the microbiota. Early adulthood to middle age in mice is generally considered a relatively stable stage. However, with aging, especially in old age as reported in previous studies, the gut microecology displays more obvious remodeling and fluctuations [70]. Studies have documented progressive structural changes in the gut microbiota with age and the systematic remodeling of the composition and function of the gut microbiota in mice at different aging stages (young-adult, middle-aged, senescent, frailty) [71]. This study selected 3-month-old and 10-month-old APOE mice as research subjects. The 3-month-old mice were in their juvenile stage, sexually mature, and phenotypically normal, serving as a baseline control; the 10-month-old mice had entered the middle age, exhibiting varying degrees of age-dependent changes in immune regulation, metabolic status, and neurological function [72]. Furthermore, previous studies have reported that 10-month-old APOE4 mice gradually develop AD-related pathological changes and cognitive impairment, and that female APOE4 mice gradually exhibit estrous cycle disorders [73–76]. Therefore, this age window is highly suitable for analyzing the individual and interactive effects of genotype, age, and sex. A systematic review study has also pointed out that the composition and functional characteristics of the gut microbiota manifest different trends with age and that β-diversity varies significantly at different developmental stages, especially in old age, indicating that the gut microbiota is more prone to fluctuations in old age and is associated with decreased activity in pathways related to carbohydrate metabolism and amino acid synthesis [77]. In this study, in the gut microbiota of 3-month-old (young) mice, the differences in α-diversity between APOE3 and APOE4 genotypes and between males and females were relatively mild. However, at 10 months of age, the microbiota richness and diversity significantly increased in APOE3 mice, while no significant changes were evident in APOE4 counterparts, suggesting that the effect of age on gut microbiota is highly associated with the APOE background and that the APOE3 mice may have stronger gut microecological adaptability or homeostasis maintenance ability in middle age.

The age-mediated effect of genotype is also reflected in exclusive genera and biomarker microbiota: the 10-month-old male and female APOE3 mice had the highest number of exclusive genera (7 each) and biomarker microbiota (10 each) across all groups, while the 3-month-old mice had only 1–6 exclusive genera and 3–6 marker microbiota across all groups. This suggests that the APOE3 genotype in middle age can induce a richer variety of specific adaptive features in the gut microbiota, which may constitute a microecological basis associated with the relatively lower AD susceptibility reported for the APOE3 genotype. Middle age may represent a critical window for exploring gut microbiota-based modulation strategies in the context of APOE-related AD susceptibility.

Furthermore, age can indirectly mediate the sex-related modification of the gut microbiota by regulating sex hormone levels, as the sex hormone levels (e.g., decreased testosterone in males and fluctuating estrogen in females) are known to change with age. This age-related change in sex hormones further exacerbated the differences in gut microbiota composition between the sexes.

### Sex as a secondary modifier of gut microbiota regulation

As a modifier of gut microbiota composition, sex does not act independently in this context, but rather depends on the dynamic action of APOE genotype and age, which is consistent with the existing reports [57]. In this study, the difference in gut microbiota diversity between male and female mice of the same genotype and age was small. However, the modifying effect of sex on the microbiota varied significantly when the genotype or age background changed. Taking the APOE3 genotype background as an example, this study observed a significant sex-age-dependent difference in the abundance of *Dubosiella*: the abundance of *Dubosiella* in 3-month-old male mice was significantly higher than in female mice of the same age, while at the 10 months of age, this trend was reversed and the abundance of this genus in female mice surpassed that in males. This dynamic change suggests that the abundance of *Dubosiella* is not only regulated by the host genetic background, but may also be closely correlated to sex- and age-related physiological conditions (such as hormone levels, metabolic state, etc.).

Previous studies have shown that sex differences can regulate the composition and metabolic function of the gut microbiota [78], which may change with age and sex hormone levels. Sex hormones (such as estrogen and testosterone) have been shown to alter the abundance of specific microbiota by affecting host immune responses, intestinal barrier integrity, and niche competition within the gut microbiota [79]. Available evidence suggests that sex may significantly affect gut microbiota composition, which can be modulated by sex hormone levels, with the gut microbiota undergoing adaptive remodeling with the changing age and hormonal status [80].

The interaction between APOE genotype and sex is more pronounced in the APOE4 context. In this study, through APOE × sex interaction analysis, the abundance of bacteria commonly associated with gut homeostasis, such as *Muribauculaceae* and *Clostridia_UCG-014*, was significantly higher in the gut of APOE4 male mice than in that of female mice. This sex-related difference suggests that APOE4 not only directly affects the composition and structure of the gut microbiota, but may also interact differently with the physiological and endocrine environments of male and female hosts, thereby dynamically regulating the homeostasis of the gut microbiota. Yet, previous studies have reported that, under non-AD-specific conditions, *Muribaculaceae* is relatively more abundant in female mice than in males, which is closely associated with sex hormone levels [81]. The disparity may be attributed to the use of different mouse models. We speculate that, within the APOE4 genetic background, *Muribaculaceae* may display an opposite sex-biased pattern, highlighting that sex-associated differences in this taxon are context-dependent and may be shaped by interactions among genetic background, disease susceptibility, and host metabolic–immune environments. The changes in the abundance of *Muribaculaceae* have been reported to be associated with phenotypes such as intestinal inflammation and mucus layer homeostasis [82]. The physiological characteristics specific to male mice (such as hormone levels and higher energy metabolism rates) may provide a suitable environment for the enrichment of this bacterial family, thus potentially contributing to the observed sex difference in its abundance. *Clostridia UCG-014* is an anaerobic bacterial community associated with host gut homeostasis. Human studies have shown that its abundance is significantly reduced in individuals with impaired intestinal barrier function, suggesting its potential association with intestinal barrier homeostasis and gut microbiota health [83]. Cross-sex fecal microbiota transplantation experiments have demonstrated that the relative abundance of *Clostridia_UCG-014* differs significantly between male and female recipients, suggesting that sex may contribute to the regulation of the distribution patterns of this taxon [84]. Based on the results of this study, it is speculated that male APOE4 mice may exhibit distinct immune or metabolic profiles (e.g., low inflammatory tendency) when compared with females, and this host physiological difference may be a key factor driving the sex-specific abundance difference in *Clostridia_UCG-014*.

### Three-way interaction of APOE genotype, age, and sex in shaping gut microbiota structure and function

This study observed that not only did the genotype, age, and sex affect the gut microbiota of APOE3/4 mice independently, their respective interactions impacted the ecological patterns of the APOE genotype animals. For example, in the analysis of the differential characteristics of the microbiota composition structure, we observed that the APOE3 and APOE4 genotypes corresponded to two distinct enterotype-like clusters dominated by *g_norank_f_Muribaculaceae* and that fine enterotype differentiation was mediated by their abundance distribution. However, cross-genus mixing was observed in the 3-month-old samples, suggesting that the gut microbiota at the 3-month-old stage are not yet have reached a fully stabilized ecological configuration and that the dual regulatory effect of APOE genotypes on “species presence + abundance distribution” is not fully established, resulting in a few samples deviating from the core cluster. In contrast, the enterotypes of the 10-month-old samples completely corresponded to the APOE genotypes, indicating that the association between APOE genotype and gut microbiota structure becomes more stable at this stage.

For example, *Allobaculum* exhibits markedly different ecological distribution patterns under different APOE genotypes. In APOE3 genotype mice, the bacterium was significantly enriched only in 3-month-old females but almost undetectable in other age or sex groups of the same genotype, demonstrating a high degree of age and sex dependence. In contrast, in APOE4 genotype mice, *Allobaculum* was stably detectable in all age and sex groups, suggesting that this taxon is consistently detectable across age and sex groups under the APOE4 background.

Furthermore, from the perspective of interaction factors, the markedly different distribution patterns of *Allobaculum* under the APOE3 and APOE4 backgrounds highlight the synergistic regulatory role among genotype, age, and sex. In the APOE3 genotype, a significant enrichment of *Allobaculum* occurred only in the 3-month-old female mice under this specific physiological condition, suggesting that its colonization may depend on the combined effects of early adulthood, sex-related endocrine environment, and relatively intact gut homeostasis. In the APOE4 genotype, however, this age and sex dependence was significantly reduced, with *Allobaculum* remaining stable in individuals of different ages and sexes. This indicates that APOE4-associated host microenvironmental differences may be associated with altered ecological thresholds for gut microbiota colonization. These results suggest that APOE genotype not only influences gut microbiota composition as a single dominant factor but also, through complex interactions with age and sex, jointly determines the timing of emergence, stability, and niche attributes of specific microbiota.

### Altered metabolic functions of the gut microbiota and their potential implications for AD

In this study, the functional prediction based on 16S rRNA gene amplicon sequencing data showed that the potential functions of gut microbiota were mainly concentrated in protein synthesis-related processes and multiple basic metabolic pathways, including translation and ribosome biogenesis, carbohydrate metabolism, amino acid metabolism, and energy metabolism. This functional profile is consistent with previous findings [85], reflecting the conserved functional attributes of the gut microbiota in supporting host nutrient metabolism.

It is noteworthy that these highly conserved metabolic and translational functions under physiological conditions often undergo remodeling in the context of diseases. Multiple studies have shown that functional shifts in gut microbiota pathways that are related to carbohydrate, amino acid, and energy metabolism may be involved in host pathological processes by influencing the production of SCFAs, energy utilization efficiency, and immune homeostasis [ 6, 86, 87]. Against this backdrop, the enrichment of the predicted metabolic pathways in this study provides a functional basis for exploring the differences in the functional potential of gut microbiota and its potential biological significance under different APOE backgrounds.

In AD-related research, alterations in gut microbiota function have increasingly been recognized as a crucial link between peripheral metabolic status and central nervous system pathology [13, 14, 88–90]. Clinical cohort and case-control studies have shown that patients with AD and individuals with MCI not only exhibit significant differences in gut microbiota composition when compared with healthy controls, but also demonstrate disturbances in microbial metabolic potential related to immune regulation, lipid metabolism, and the synthesis of neuroactive substances [55, 88, 89]. These studies suggest that the role of gut microbiota in AD may be more reflected in changes in its overall metabolic functional network, rather than simply the increase or decrease of a single genus. The functional prediction results revealed in this study, focusing on metabolic pathways and protein synthesis, echo the “metabolic imbalance” emphasized in the aforementioned AD-related studies on the functional level.

Animal experiments further support a potential contributory role of gut microbiota metabolism in AD pathology. Germ-free AD model mice have shown significantly reduced Aβ deposition levels, while gut microbiota reimplantation could restore or exacerbate amyloid pathology, accompanied by enhanced neuroinflammation and cognitive impairment [90, 91]. Mechanistic studies have shown that gut microbiota can influence the host immune-metabolic axis through SCFAs, bile acid metabolites, immunomodulatory factors, and various neuroactive metabolites. These pathways are closely related to core pathological processes in AD, such as chronic neuroinflammation, abnormal phosphorylation of tau protein, and neuronal energy metabolism imbalance [92]. The functional prediction results of this study seem to indicate that, under different APOE backgrounds, the link between the potential changes in metabolic function and the structural differences of gut microbiota may provide functional clues linking peripheral microecological alterations to AD-related pathological processes.

### Potential mechanisms and clinical implications

Based on the functional changes inferred by functional prediction analysis in this study, the interaction of APOE genotype, age, and sex in regulating the gut microbiota may involve multiple interrelated mechanistic pathways through the gut-brain axis. Gut microbiota imbalance can may contribute to peripheral and central inflammatory responses through the “intestinal barrier-inflammation axis”. On the one hand, pathogen-associated molecular patterns from microorganisms, such as lipopolysaccharide and peptidoglycan, can activate innate immune signaling pathways [93, 94]. Meanwhile, impaired intestinal barrier function can lead to increased permeability (“leaky gut”), thus making it easier for these microbial products to enter the circulatory system and promoting the development of chronic low-grade inflammation [95, 96]. In this study, we observed an increased proportion of potentially pathogenic bacteria and anaerobic bacteria in APOE4 mice, which is consistent with a microbiota profile associated with dysbiosis and a pro-inflammatory tendency, thereby potentially amplifying the aforementioned inflammatory process [97]. As we age, the intestinal barrier function and immune regulation capacity gradually decline, which may promote the spread and persistence of inflammation-related signals, thereby aggravating the overall inflammatory state [98–100]. Meanwhile, sex differences may influence the host’s response to inflammatory signals through hormone-mediated immune regulation mechanisms, thereby leading to differentiated inflammatory response patterns among different individuals [101]. Ultimately, these changes may promote microglial activation and maintain a chronic neuroinflammatory state, thereby potentially linking gut microbiota dysbiosis to AD-related neuropathology [102].

Microbial metabolites serve as crucial mechanistic links between the gut microbiota and host physiological functions. SCFAs, bile acids, and tryptophan metabolites can regulate immune homeostasis, energy metabolism, and neural signaling pathways [103–106]. Functional prediction analysis revealed that gut microbiota function was primarily enriched in carbohydrate, amino acid, and energy metabolism-related pathways, suggesting that metabolic-related function plays a prominent role in the overall microbiota function. Given the microbiota structure and functional phenotype results, it is speculated that the metabolic output patterns of the gut microbiota may vary under different genotype backgrounds, thereby affecting the balance between pro-inflammatory and anti-inflammatory responses and further impacting neural-related processes, including synaptic plasticity and the neurotransmitter system. Simultaneously, age-related metabolic reprogramming may further impact the overall state of microbiota metabolic function [27], while the sex hormone-dependent regulation may also lead to differences between sexes in microbial metabolism and its effects on the gut-brain axis [80].

Notably, the host genetic background plays a crucial regulatory role in the aforementioned processes. The APOE genotype, especially APOE4, is closely associated with enhanced inflammatory responses, impaired lipid transport, and decreased neural repair capacity [17, 21, 107]. These factors may influence the composition and function of the gut microbiota by altering intestinal immune status and the metabolic environment, which is consistent with our observation that APOE4 mice exhibited a declining trend in microbiota diversity with age, whereas APOE3 mice maintained relatively stable diversity. Furthermore, age-related immunosenescence and sex hormone-dependent regulation may jointly affect the host-microbiota interaction [27, 78]. Our results showed that APOE3 mice maintain a high microbiota diversity with age, while APOE4 mice exhibit a declining diversity trend, suggesting that genotype-age interaction may play a key role in maintaining microbiota homeostasis, with sex exerting a modifying effect on this.

From a clinical perspective, this study provides experimental evidence for understanding the synergistic effects of host genetic factors, age, and sex on regulating the gut microbiota. Identifying gut microbiota functional characteristics on the basis of APOE genotype, age, and sex may help improve the risk stratification for AD and facilitate the early identification of high-risk individuals. Furthermore, given the modifiable nature of the gut microbiota, these results highlight the potential for developing personalized intervention strategies based on individual genetic background, age, and sex characteristics, such as dietary regulation, probiotic application, or fecal microbiota transplantation, thereby achieving precise intervention. Simultaneously, microbiota-related functional characteristics hold a great promise as non-invasive biomarkers for early disease screening and dynamic monitoring. Therefore, combining microbiome data with genetic information and clinical indicators may improve the accuracy of AD prediction models. Targeting the gut-brain axis may also become an important therapeutic strategy for regulating neuroinflammation, restoring metabolic homeostasis, and slowing disease progression.

In summary, this study provides an experimentally supported theoretical framework at the level of potential mechanisms, based on our findings, revealing how host genetic background, age, and sex may jointly participate in AD-related processes by regulating gut microbiota function. The findings may lend support to the integration of microbiome strategies into precision prevention and treatment systems.

## Limitations and future directions

While this study provides preliminary evidence for the synergistic regulatory effects of APOE genotype, age, and sex on the gut microbiota, it still has the following limitations: First, the relatively limited sample size (*n* = 7–10 per group) may affect the statistical stability of the three-factor interaction analysis, especially in assessing the modifying effects of sex and age. Second, data based on 16S rRNA gene amplicon sequencing can only be resolved to the genus level, which limits the ability to distinguish functional differences at the strain level, while different strains within the same genus may exhibit significant heterogeneity in metabolism and immune regulation. Third, this study has not directly detected gut microbiota-related metabolites, intestinal barrier integrity, and peripheral immune indicators, limiting the direct validation of the impact of microbiota changes on host physiological functions. Fourth, the regulatory mechanisms underlying the combined effects of APOE genotype, age, and sex on key signaling molecules of the gut-brain axis, such as neurotransmitters and inflammatory mediators, remain to be systematically elucidated. Importantly, this study is primarily observational and lacks direct intervention-based experiments to establish causal relationships between gut microbiota alterations and AD-related phenotypes. Therefore, the conclusions regarding the functional roles of microbiota remain associative rather than causative. Fifth, this study only selected two time points, 3 months and 10 months of age, which is insufficient to reflect the dynamic evolution of the gut microbiota during aging. Sixth, the study mainly focused on changes in the gut microbiota and has not yet systematically evaluated its direct association with the core pathological phenotypes of AD. Finally, the results from the mouse model still need to be interpreted with caution when extrapolated to the human population.

To address the aforementioned limitations, future research is awaited at several levels. First, it is necessary to systematically analyze the time-dependent characteristics of APOE genotype, age, and sex on gut microbiota regulation by expanding the sample size and introducing multi-timepoint or longitudinal tracking designs. Second, combining metagenomic sequencing and metatranscriptomics can help reveal the fine mechanisms of the three-factor synergistic regulation at the strain and functional gene levels. Furthermore, incorporating gut metabolomics, intestinal barrier function assessment, and peripheral immune phenotype analysis into the research framework can more directly verify the impact of microbiota changes on host physiological functions and refine the evidence chain linking genotype, age, sex, gut microbiota alterations, and AD pathology. Importantly, future studies should incorporate interventional experimental designs with causal inference capabilities. For example, fecal microbiota transplantation from APOE4 or aged mice into germ-free or antibiotic-treated recipient mice could be performed to assess whether microbiota alterations are sufficient to induce AD-related phenotypes. Simultaneously, targeted microbiota intervention strategies, such as antibiotic depletion or probiotic supplementation, may be applied to assess whether reversing specific microbial changes can ameliorate neuroinflammation and cognitive impairment. These approaches will contribute to the exploration of causal links between gut microbiota composition and disease-related phenotypes. In addition, combining core AD pathological indicators (such as Aβ deposition and tau pathology) with cognitive behavioral assessments will help elucidate the pathological significance of gut microbiota alterations. Finally, it is necessary to combine animal model studies with human cohort studies in the future to assess the clinical relevance and potential translational value of the findings in this study.

## Conclusion

This study, using APOE3 and APOE4 genotype mouse models, systematically evaluated the interactive influences of age and sex on gut microbiota structure and potential function. The results indicate that gut microbiota variation cannot be explained by a single factor, but rather by the combined impact from the interaction of APOE genotype, age, and sex. Different combinations of factors exhibit specific microbiota characteristics and functional prediction patterns.

This study expands the analytical framework of gut microbiota in AD-related research from a multi-factor interaction perspective, emphasizing the importance of incorporating age and sex stratification when analyzing APOE4-related risk. Overall, this study provides new microecological evidence for understanding the associations between peripheral gut microbiota alterations and APOE4-related AD susceptibility, and offers a conceptual framework for future individual-stratified microecological intervention studies.

## Electronic Supplementary Material

Below is the link to the electronic supplementary material.

Supplementary Material 1: Table S1 PERMDISP analysis of gut microbiota β-diversity under different distance metrics

Supplementary Material 2: Table S2 Differentially abundant gut microbiota taxa among APOE genotype × age × sex groups

Supplementary Material 3: Table S3 Differentially abundant gut microbiota taxa between APOE3 and APOE4 mice

Supplementary Material 4: Table S4 Differentially abundant gut microbiota taxa among APOE genotype × age groups

Supplementary Material 5: Table S5 Differentially abundant gut microbiota taxa among APOE genotype × sex groups

Supplementary Material 6: Table S6 Predicted gut microbiota phenotypes based on BugBase analysis

Supplementary Material 7: Table S7 Predicted functional categories of gut microbiota based on COG analysis

Supplementary Material 8: Table S8 Predicted KEGG pathways of gut microbiota at Level 2

Supplementary Material 9: Table S9 Predicted KEGG pathways of gut microbiota at Level 3

## Data Availability

The raw 16S rRNA gene sequencing data generated in this study have been deposited in the NCBI SRA under the BioProject accession number PRJNA1438919. The data are publicly available via the NCBI database.

## Abbreviations

AD: Alzheimer’s disease
APOE: Apolipoprotein E
ASV: Amplicon sequence variant
Aβ: Amyloid-β
FDR: False discovery rate
JSD: Jensen-Shannon divergence
LEfSe: Linear discriminant analysis effect size
MCI: Mild cognitive impairment
NMDS: Non-metric multidimensional scaling
NCBI: National Center for Biotechnology Information
PCoA: Principal coordinates analysis
PCR: Polymerase chain reaction
PERMDISP: Permutational analysis of multivariate dispersions
PERMANOVA: Permutational multivariate analysis of variance
SCFAs: Short-chain fatty acids
SRA: Sequence read archive
